# Assessment of addictive behaviors in patients with schizophrenia

**DOI:** 10.1192/j.eurpsy.2023.1394

**Published:** 2023-07-19

**Authors:** M. Kacem, W. Bouali, H. Babba, F. Zaouali, L. Zarrouk

**Affiliations:** Psychiatric department, Taher Sfar Hospital of Mahdia, Mahdia, Tunisia

## Abstract

**Introduction:**

Schizophrenia, a chronic and complex psychiatric pathology, can be isolated. However, it can be associated with other comorbidities and thus be accompanied by addictive behaviors that complicate their management.

**Objectives:**

The objectives of our study were to estimate the prevalence and identify the characteristics of addictive behaviors in patients with schizophrenia.

**Methods:**

A retrospective study of 151 patients with schizophrenia hospitalized in the psychiatry department of Taher Sfar University Hospital in Mahdia from January 2017 to December 2021.

**Results:**

The mean age of the patients was 39.8 ± 11.23 years, with a predominance of the 36-45 age group (38.4%). All patients were male. Three quarters of the patients (75.5%) were users of psychoactive substances (PAS): nearly three quarters (72.8%) were addicted to tobacco, more than one third (39.7%) were addicted to alcohol, more than one quarter (29.1%) were addicted to cannabis and nearly one quarter (26.5%) were addicted to other PAS. In more than half of the cases (54.4%), the age of onset of substance use was between 16 and 25 years. The use of PAS preceded the onset of schizophrenia in 62.3% of cases. The relationship with the entourage was marked by hetero-aggressiveness in 77.5% of patients, withdrawal from the entourage in 16.6% of patients and conflict in 5.3% of patients. The impact on the relationship with oneself was marked by self-aggressiveness in 18.5% of patients. Concerning the professional impact, three quarters of the patients (76.1%) had to stop working. The majority of patients (84.1%) continued their usual treatment, while 15.2% of patients stopped it. Only one patient required an increase in dose.

**Conclusions:**

Subjects suffering from schizophrenia are particularly vulnerable to addictions, mainly to tobacco and alcohol. They are thus a group more at risk of the deleterious effects of psychoactive substances and of the aggravation of the clinical and psychosocial evolution of their psychiatric disorders. Measures for early detection and treatment of their addictive behaviors even before the onset of schizophrenia should be proposed.

**Disclosure of Interest:**

None Declared

